# Predictors of malignancy in pancreatic head mass: a prospective study

**DOI:** 10.4314/pamj.v9i1.71206

**Published:** 2011-07-19

**Authors:** Arjun Sivarman, Ashok Muthukrishnan, Nagendra Boopathy Senguttvan, Suraj Anil Suchak, Umashankkar Kannan

**Affiliations:** 1 Department of Surgery , Kasturba Medical College, Manipal, Karnataka, India; 2 Department of Cardiology, All India Institute of Medical Sciences, New Delhi, India; 3 Department of Surgery, All India Institute of Medical Sciences, New Delhi, India

**Keywords:** Pancreatic Carcinoma, CA 19-9 Antigen

## Abstract

**Introduction:**

The objective of the study was to identify the predictive factors for malignancy in pancreatic head mass as a primary outcome and assess the value of CA 19-9 as a diagnostic tool for malignancy as a secondary outcome.

**Methods:**

A prospective study of patients presented with pancreatic head mass was conducted in a tertiary care referral hospital, Manipal, India from May 2006 to November 2008. The study population was divided into malignant and benign groups based on the final histopathology report. A univariate and multivariate analysis of potential predictive factors for malignancy were conducted.

**Results:**

A total of 102 patients with pancreatic head mass were included in the study after fulfilling the inclusion/exclusion criteria. 78 were malignant and 24 were benign. There was significant weight loss (p<0.001) and high mean bilirubin levels (p=0.002) in the malignant group. Mean CA 19-9 was significantly higher in the malignant group (290.7 vs. 30.3 U/ml; p<0.001). Sensitivity and specificity of CA 19-9 for detecting malignancy in pancreatic head mass at a cut off of 35U/ml was 86% and 79% respectively. CA 19-9 positivity rate was higher with increasing cut off values of 100, 200 and 300U/ml but such high levels occurred in fewer patients. All the non-jaundiced patients (100%) with raised CA 19-9 levels were found to be malignant compared to 86% malignancy in jaundiced patients. In multivariate analysis, a combination of weight loss>10% of body weight and bilirubin>3 mg/dl and CA 19-9>35U/ml had specificity and positive predictive value of 100% for predicting malignancy in pancreatic head mass.

**Conclusion:**

The presence of weight loss and jaundice and raised CA 19-9 levels together in a patient with pancreatic head mass can be predictive of malignancy. A very high CA 19-9 level can be an indicator of malignancy in a pancreatic head mass. A raised CA 19-9 level may be more predictive of malignancy in non-jaundiced patients than in jaundiced patients.

## Introduction

Pancreatic carcinoma accounts for only 2.6% of all newly diagnosed malignancies [1,2]. It is the ninth most common malignancy and fifth most common cause for cancer related deaths [3]. The overall 5-year disease-free survival rate is on the order of 1% to 2% [4]. Only 10% of patients have localized disease on presentation. Survival, however, is increased in those patients undergoing resection compared with those not undergoing surgery regardless of stage [5,6].

The morbidity and mortality associated with pancreatic head resections are relatively high when compared with distal pancreatic resections because of the anatomical location of the head of pancreas which predisposes to infiltrate adjacent important structures. With the benign pancreatic masses closely mimicking malignancy, especially the chronic pancreatitis-inflammatory mass, accurate identification of malignancy in a pancreatic head mass is of utmost importance for early diagnosis and aggressive treatment of this lethal disease and also in avoiding a radical resection for a benign mass. We conducted a study to find the possible predictors of malignancy in a pancreatic head mass using clinical and biochemical parameters.

## Methods

### Study design

A prospective study was conducted in Kasturba Hospital, Manipal, India from May 2006 to November 2008. The study group constituted patients admitted with a radiological diagnosis of pancreatic head mass. CA19-9 levels and a tissue diagnosis were obtained in all the patients. All the patients underwent Endoscopic retrograde cholangiopancreatogram (ERCP) brushings/cytology or/and Endoscopic ultarsonogram (EUS) -guided fine needle aspiration cytology (FNAC) as initial evaluation. This protocol was followed for a better loco-regional staging of the disease and also to identify conditions like lymphoma, endocrine tumors etc. that does not require radical pancreatic resections. The cytology/FNAC report divided the patients into one of the four arms -malignant, suspicious of malignancy, inconclusive, benign. All patients with radiologically resectable mass and FNAC reported as malignant/suspicious of malignancy/inconclusive underwent surgical exploration and resection. Patients with radiologically unresectable mass and FNAC report as suspicious of malignancy and inconclusive were subjected to repeat FNAC. Lesions that failed to yield any results on repeat FNAC were subjected to open biopsy. All the patients in the benign arm were followed up by regular clinical visits and repeated imaging for a minimum of 12 months and those presented with progression of the disease were considered malignant and evaluated for resection. Patients with cystic neoplasm, mass body and tail of pancreas, endocrine tumors, recurrent tumors and, peri-ampullary tumors (Ampulla of Vater, Duodenal carcinoma, Distal CBD) were excluded to improve the validity of our result to solid pancreatic head mass alone. Patients in benign and malignant groups were compared for possible predictors of malignancy

### CA 19-9 measurement

CA 19-9 was measured in 3ml of fresh serum obtained from peripheral venous sample. The kit used was ELISA for CA19-9 (Omega diagnostics co. Ltd., United Kingdom). The recommended cutoff value was 35 U/ml.

### Statistical analysis

Patient demographics, risk factors, associated symptoms including weight loss, jaundice and CA 19-9 values were the variables compared between the groups. Multivariate analysis of the variables was also performed to explore the possible combinations of variables that could predict malignancy.

### Statistical analysis

Patient demographics, risk factors, associated symptoms including weight loss, jaundice and CA 19-9 values were the variables compared between the benign and malignant groups. Multivariate analysis was further performed for those variables that were significantly different between the two groups to explore a possible combination of variables could predict malignancy. Self reported or documented weight from prior medical visits was used in the estimation of weight loss. This may be subjected to recall error and subsequent error in calculation of the actual weight loss. However, self-reported weight has been shown to be accurate [7] and most epidemiological studies have used self-reported weight and weight change data [8,9]. Student – t test was used for comparing means of independent samples like age and weight loss. Chi square test was used to compare proportion like those of sex and weight loss. Wilcoxon rank sum test was used to compare means of ordinal variables like bilirubin and CA 19–9 levels.

Weight loss, jaundice and CA 19-9 levels were significantly different between the two groups and were further analyzed to find the optimal cut off that can predict malignancy with maximum yield. Conventional definition of clinically significant loss of more than 10% of body weight was used as a cut off for weight loss [10]. A Receiver Operating Characteristic (ROC) curve was used in the estimation of cut off values for Jaundice and CA 19-9 levels. ROC curve is a commonly used and widely accepted statistical tool to establish cut off levels for a test [11]. Briefly, ROC curve is generated by plotting 1-specificity along x-axis and sensitivity along y-axis at different interpretation thresholds for a particular test. An ideal diagnostic test characterized by high true and low false positive rates will have a ROC curve upwards and to the left up corner close to the x-axis. In our study, cut off for bilirubin and CA 19-9 levels obtained by ROC were 3mg/dl and 35U/ml respectively.

Further, Multivariate analysis was performed comparing the combination of weight loss more than 10%, bilirubin more than 3 mg/dl and CA 19 –9 levels more than 35 U/ml between two groups. p-value<0.05 is considered significant. Data analysis was done using SPSS software 11.0.1 version.

## Results

### Mode of tissue diagnosis

Of 127 patients that were admitted during the study period, 102 patients were enrolled after fulfilling the inclusion criteria. After the initial ERCP brushings/cytology or/and EUS -guided FNAC, study population was divided into 51 malignant (group 1), 23 suspicious of malignancy (group 2), 13 inconclusive (group 3) and 15 benign patients (group 4). Resection was attempted in 24 patients with radiologically resectable mass, of which 16 cases had pre-operative report as malignant and 5 were suspicious of malignancy and 3 had an inconclusive report. Intraoperatively, 20 patients underwent Whipple's pancreatico-duodenectomy and their final histopathology report of the resected specimen in four of the twenty patients showed chronic pancreatitis with benign inflammatory mass while the remaining 16 cases were malignant. Of the four benign cases that underwent resection, three were preoperatively reported as suspicious of malignancy and one as inconclusive. All the 16 malignant cases were preoperatively reported as malignant. Four of the 24 patients who underwent resection were unresectable and hence surgical bypass and biopsy was performed. Final histopathology report of all the four unresectable mass was malignant. Repeat FNAC was performed in the remaining 18 cases in group 2 and 10 cases from group 3 with radiologically unresectable mass. After repeat FNAC, malignancy was reported in 15 out of 18 cases from group 2 and 4 out 10 cases from group 3 and four patients from group 3 were reported as benign. Remaining 3 cases from group 2 and two cases from group 3 were still inconclusive underwent open biopsy. Open biopsy revealed malignancy in 4 out of 5 cases and one case from group 3 was reported as benign. Ten patients in malignant arm with unresectable mass underwent surgery for drainage procedure as endoscopic drainage was unsuccessful / failed and a biopsy was performed during the surgery. None of the cases which were initially negative for malignancy in EUS guided FNAC presented with signs of malignancy on review during 12 months of follow up. Table 1 shows the mode of tissue diagnosis for all the patients included in the study.

**Table 1:** Mode of tissue diagnosis for all the patients included in the study

Mode of diagnosis	Malignant	Benign
Resection/Open Biopsy	31	5
Fine Needle Aspiration Cytology (FNAC)/Cytology	47	19

### Single variable analysis

In this study, the incidence and mean value of weight loss was significantly higher in the malignant group. The mean bilirubin and the CA19-9 levels between the two groups were significantly higher in the malignant group (table 2). Mean CA 19-9 levels in patients with biliary obstruction was higher than patients without obstruction irrespective of benign or malignant mass (table 3). Demographics did not yield any statistical difference between two groups. Smoking and alcohol intake were present almost in equal proposition in both the groups. No statistical difference was noted in the presence of associated diabetes mellitus, family history of pancreatic cancer, associated pain abdomen or presence of malabsorption.

**Table 2:** Variables that were compared between the two groups

Variable	Malignant	Benign	P Value
Age (years)	56.86 +/- 10.7	57.75 +/-15.2	0.810
Proportion of male (%)	77.8	56.3	0.18
Mean percent of weight loss (%)	17.4	4.3	<0.001
Proportion of weight loss (%)	83.3	12.5	<0.001
Mean bilirubin (mg/dl)	9.3	1.7	0.002
CA 19-9 (u/ml)	290.8	30.3	<0.001

**Table 3:** Mean CA19-9 levels in malignant and benign patients with and without obstruction

Mean ca 19-9 levels (u/ml)	Malignant	Benign
With biliary obstruction	402.9	47.5
Without biliary obstruction	178.7	13.1

### Receiver Operating Characteristics (ROC) Curve and cut off values

**Figure 1:**
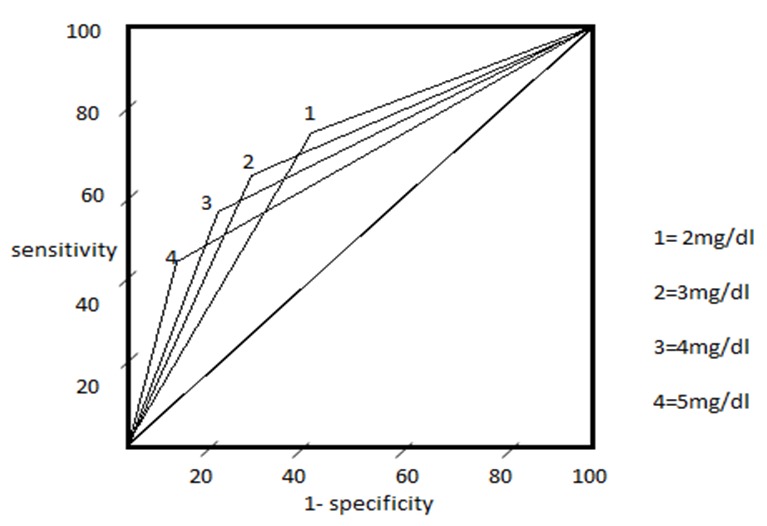
Receiver Operating characteristic (ROC) curves for different values of Bilirubin

**Figure 2:**
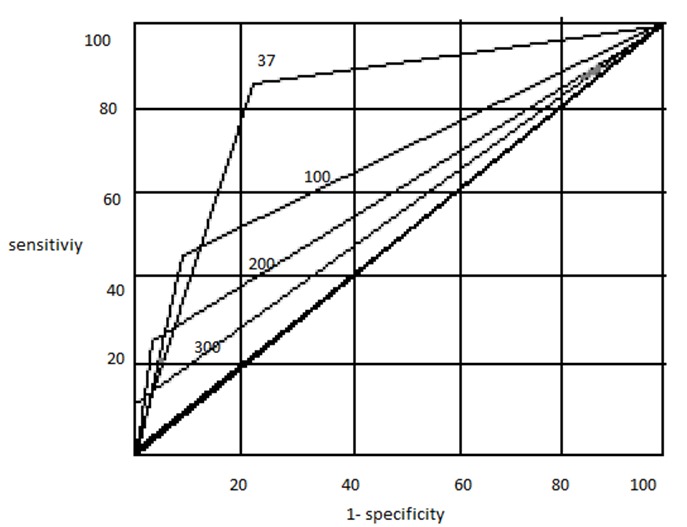
Receiver Operating characteristic (ROC) curve for different values of CA 19-9

Receiver Operating Characteristic (ROC) curve was employed for obtaining optimal cut off values for jaundice and CA 19-9 levels. ROC curve using different cut off values of these variables revealed a trade of between sensitivity and specificity for predicting malignancy. Figure 1 and 2 shows the ROC curves for these variables at different cut off values and it clearly indicates the optimal threshold for bilirubin level was 3mg/dl and CA 19-9 was 35U/ml.

The sensitivity, specificity of these variables for predicting malignancy when used alone was studied (table 4). CA 19-9 had the highest sensitivity (86%) of all the other variables. Sensitivity and specificity of CA19-9 at different cut off levels are shown in table 5. None of the three variables were effective in predicting malignancy individually.

**Table 4:** Univariate analysis of the three predictors studied

Variable	Sensitivity %	Specificity %	PPV %	NPV %
Weight loss (>10% in six months)	83	88	96	62
Jaundice (>3mg/dl)	61	71	87	38
CA 19-9 (>35U/ml)	86	79	93	63

**Table 5:** Sensitivity and Specificity of CA 19-9 in different cut off levels

CA 19-9 cut off levels (U/ml)	Benign		Malignant		Sensitivity %	Specificity %
			
	Normal	Increased	Normal	Increased		
**37**	19	5	11	67	86	79
**100**	21	3	46	32	41	87
**200**	23	1	60	18	23	96
**300**	24	0	71	7	8	100

### Jaundice and CA 19-9 levels

Out of 72 patients with raised CA 19-9 levels, 43 were found to have jaundice while 29 were non-jaundiced. It was observed that all the 29 non-jaundiced patients were malignant whereas 6 out 43 jaundiced patients were benign. Proportion of malignancy in jaundiced patients with raised CA 19-9 levels was 86% and Proportion of malignancy in non-jaundiced patients with raised CA 19-9 levels was 100%. Moreover CA 19-9 positivity in malignant jaundiced patients was 97% and in non-malignant jaundiced patients was 85%.

### Multivariate analysis

Sensitivity and specificity for predicting malignancy when any one/two variable/s was present in a patient is shown in table 6. Multivariate analysis demonstrated that all the 35 patients who had three variables present were malignant while none of the 24 benign patients had all the three variables present indicating a specificity and positive predictive value of 100% for detecting malignancy in pancreatic head mass (Table 6). Radiological evidence of malignancy was noted either in CT or ERCP or EUS in 32 out of 35 patients with all three variables present and none of the 24 benign patients. Hence when radiological evidence of malignancy was included in the multivariate analysis sensitivity and negative predictive value was reduced to 41% and 34% (Table 6).

**Table 6:** Multivariate analysis of the three predictors studied

Patients with pancreatic mass	Sensitivity (%)	Specificity (%)	Positive predictive Value (%)	Negative predictive Value (%)
Weight loss>10% or bilirubin>3 mg/dl or raised CA 19-9 level>35U/ml	94	54	87	72
Combination of any two variables present	71	67	92	41
Weight loss>10% and bilirubin>3 mg/dl and raised CA 19-9 level >35U/ml with radiological evidence of malignancy	41	100	100	34
Weight loss>10% and bilirubin>3 mg/dl and raised CA 19-9 level >35U/ml	45	100	100	36

## Discussion

### Univariate and Multivariate analysis

In our study demographics were comparable between the benign and malignant groups. Apart from most of the patients (76/102) with a documented weight in prior hospital visits, we employed a self reported weight six months prior to the diagnosis of the mass in few patients (26/102). Weight loss, jaundice and CA 19-9 levels were the variables that were significantly higher in the malignant group.

We used ROC curve for the statistical analysis of bilirubin and CA 19-9 levels ROC curve has been extensively used by Hong-Ja Kim et al in their statistical analysis of CA 19-9 in various pancreatico-biliary diseases [12]. In our study, cut off for bilirubin and CA 19-9 levels obtained by ROC were 3mg/dl and 35U/ml respectively. Weight loss and CA 19-9 levels had almost similar sensitivity and specificity with jaundice with the lowest values. However these variables cannot be used individually to predict malignancy as their sensitivity and specificity were low. But when these three variables are present together in a patient with pancreatic head mass the specificity, positive predictive value were 100% for predicting malignancy. As far as our knowledge, Tessler et. al [13] were the first to correlate the clinical and biochemical parameters in combination to increase the efficiency of differentiating the malignant from benign lesions without a tissue diagnosis and their associated complications. A total of 150 patients were studied by them, and established that weight loss and bilirubin levels were significantly higher in the malignant group. In multivariate analysis, a combination of weight loss >20 lbs, bilirubin >3 mg/dl, and CA 19-9 >37 U/ml had both a specificity and positive predictive value of 100% for predicting malignancy. His study was a retrospective study based on data collected on patients operated for suspected pancreatic malignancy, which includes patients with pancreatic mass (head, body and tail) and biliary stricture also. In our study we have narrowed our patients to only pancreatic head mass, which is a common clinical scenario – pancreatic head mass – benign or malignant? The mean CA 19-9 levels in malignant and a benign group in our study was higher than Tessler et. al patients. This observation is possibly because Tessler has included body and tail mass, which are less likely to present with biliary obstruction and hence a lower value of CA 19-9 levels. Moreover CA 19-9 levels in our study are taken before biliary drainage of any form was attempted.

### CA 19-9 and Jaundice

Other interesting observation of the study was the significance of raised CA 19-9 levels in jaundiced patients and non jaundiced patients with head mass. All the patients with raised CA 19-9 levels without jaundice were malignant whereas only 86% of the patients with jaundice were malignant. This implies the significance of raised CA 19-9 levels in non-jaundiced patients than in jaundiced patients with pancreatic head mass. CA 19-9 antigen is synthesized both by the epithelial cells of the normal biliary tract and by the tumor cells and excreted within the bile [14]. It is suggested that the CA 19-9 antigen, which is high in concentration in the bile of the patients with benign and malignant obstructive jaundice, refluxes into the bloodstream due to the increase in the permeability between bile and blood, secondary to the bile stasis; moreover, it is stated that there can be an inability to degrade the antigen in the liver due to a hepatic dysfunction [15]. In the absence of obstructive jaundice the CA 19-9 present in the blood is exclusively by the tumor cells excluding the false positive possibility from the biliary tract epithelium. Hence a patient with pancreatic head mass without jaundice and raised CA 19-9 levels are more likely to be malignant than the jaundiced patients. Nuvit Duraker et al [16] in their study compared the CA 19-9 levels between benign and malignant pancreatic mass with and without jaundice also observed the similar importance of raised CA 19-9 levels in non-jaundiced patients. However, this observation did not attain statistical significance in our study and it requires further studies with more patients and carefully selected cohorts.

### CA 19-9 – Predictive Value

Sensitivity and specificity of CA 19-9 for predicting malignancy in a patient with pancreatic head mass in our study was 86 and 79 respectively when the cut off value was 35U/ml. The values were comparable with other studies as shown in table 7. When the cut off values were raised to 100, 200 and 300U/ml the positivity rate and specificity of CA 19-9 increased but such high values were seen only in fewer patients. At CA 19-9 value of 300U/ml the specificity and positive predictive value were 100% for predicting malignancy (Table 5). Though there are isolated case reports of very high CA 19-9 levels in benign conditions [17] we can still consider it as an independent predictor of malignancy in the setting of the pancreatic mass lesion. This finding was also confirmed by Bedi et al in their study on mass lesions of chronic pancreatitis [18]. However, studies dedicated exclusively on very high CA 19-9 levels will help us in better understanding of this issue.

**Table 7:** Comparison of the predictive value of CA19-9

Author	Present Study	Nuvid Duraer et Al [16]	Cwik G et Al [19]	Goonetillek E Ks et Al [20]
Year	2009	2007	2006	2007
Type of the study	Prospective	Prospective	Prospective	Systematic Review
Sensitivity (%)	86	81	81	79
Specificity (%)	79	76	89	82
Positive predictive Value (%)	93	77	93	81
Negative Predictive Value (%)	63	80	89	80

## Conclusion

The clinical researches for the management of pancreatic carcinoma has been ever increasing to achieve early detection and deliver appropriate radical treatment to prolong the survival and also reducing the number of patients with benign mass undergoing inadvertent radical resection. Attempts are always being made in all possible ways to brighten this grey area – pancreatic head mass – benign or malignant? In our attempt we found that, under appropriate clinical circumstances, in a patient with pancreatic head mass, presence of weight loss and jaundice and raised CA 19-9 levels can be predictive of malignancy and a radical resection should be considered even if the pre operative tissue diagnosis is not available. Very high CA 19-9 levels, though have occasionally been associated with benign conditions, were more specific for malignancy in the setting of a pancreatic head mass. Also, elevated levels of CA 19-9 have been found to predict malignancy better in non-jaundiced patients than in jaundiced patients but without reaching statistical significance. As our study was a retrospective review of prospectively selected cohort of patients, further prospective studies are required to validate the results of the study. In spite of these advances, the principle of pancreatic head mass management should still be radical resection for all pancreatic mass with suspicion of malignancy or inconclusive reports. The concerns of procedural morbidity and mortality for pancreatic mass that may ultimately be proved benign should not delay a radical resection for mass with suspicion of malignancy or inconclusive reports.
