# Erratum: predictors of malignancy in pancreatic head mass: a prospective study

**DOI:** 10.11604/pamj.2016.23.104.9256

**Published:** 2016-03-16

**Authors:** The Pan African Medical Journal

**Affiliations:** 1 The Pan African Medical Journal, Kampala, Uganda

**Keywords:** Erratum, pancreatic carcinoma, CA 19-9 antigen

## Abstract

This erratum corrects “Predictors of malignancy in pancreatic head mass: za prospective study”. The Pan African Medical Journal. 2011;9:30.

## Erratum

The original version of this article [1] contained a typographical error: an author’s name was misspelt. The error has been corrected in both the PDF and HTML versions of the article. The author’s name has been changed to: Arjun Sivaraman.
